# The LSD1-Interacting Protein GILP Is a LITAF Domain Protein That Negatively Regulates Hypersensitive Cell Death in *Arabidopsis*

**DOI:** 10.1371/journal.pone.0018750

**Published:** 2011-04-19

**Authors:** Shanping He, Guihong Tan, Qian Liu, Kuowei Huang, Jiao Ren, Xu Zhang, Xiangchun Yu, Ping Huang, Chengcai An

**Affiliations:** The State Key Laboratory of Protein and Plant Gene Research, College of Life Sciences, Peking University, Beijing, China; University of South Florida College of Medicine, United States of America

## Abstract

**Background:**

Hypersensitive cell death, a form of avirulent pathogen-induced programmed cell death (PCD), is one of the most efficient plant innate immunity. However, its regulatory mechanism is poorly understood. AtLSD1 is an important negative regulator of PCD and only two proteins, AtbZIP10 and AtMC1, have been reported to interact with AtLSD1.

**Methodology/Principal Findings:**

To identify a novel regulator of hypersensitive cell death, we investigate the possible role of plant LITAF domain protein GILP in hypersensitive cell death. Subcellular localization analysis showed that AtGILP is localized in the plasma membrane and its plasma membrane localization is dependent on its LITAF domain. Yeast two-hybrid and pull-down assays demonstrated that AtGILP interacts with AtLSD1. Pull-down assays showed that both the N-terminal and the C-terminal domains of AtGILP are sufficient for interactions with AtLSD1 and that the N-terminal domain of AtLSD1 is involved in the interaction with AtGILP. Real-time PCR analysis showed that *AtGILP* expression is up-regulated by the avirulent pathogen *Pseudomonas syringae pv. tomato* DC3000 *avrRpt2* (*Pst avrRpt2*) and fumonisin B1 (FB1) that trigger PCD. Compared with wild-type plants, transgenic plants overexpressing AtGILP exhibited significantly less cell death when inoculated with *Pst avrRpt2*, indicating that AtGILP negatively regulates hypersensitive cell death.

**Conclusions/Significance:**

These results suggest that the LITAF domain protein AtGILP localizes in the plasma membrane, interacts with AtLSD1, and is involved in negatively regulating PCD. We propose that AtGILP functions as a membrane anchor, bringing other regulators of PCD, such as AtLSD1, to the plasma membrane. Human LITAF domain protein may be involved in the regulation of PCD, suggesting the evolutionarily conserved function of LITAF domain proteins in the regulation of PCD.

## Introduction

Plants have evolved two branches of innate immune system to protect themselves against pathogen attack [1]. Plants recognize pathogen-associated molecular patterns (PAMPs) through transmembrane pattern recognition receptors (PRRs) to initiate PAMP-triggered immunity (PTI), which acts as the first line of defense [1]. Effector-triggered immunity (ETI) serves as the second line of defense and is one of the most efficient innate immunity in plants. ETI involves both direct and indirect recognition of pathogen effectors by plant resistance (R) proteins and usually leads to a hypersensitive cell death response (HR), which is defined as rapid and localized plant cell death at the infection site [2]. For example, the *Arabidopsis* R protein RPS2 recognizes the avrRpt2 effector expressed by *Pseudomonas syringae pv. tomato* DC3000 *avrRpt2* (*Pst avrRpt2*) to trigger hypersensitive cell death [3], [4]. Although hypersensitive cell death is the most extensively investigated form of plant programmed cell death (PCD) [5], its regulatory mechanism is poorly understood.

To identify regulators of hypersensitive cell death, investigators have isolated a number of lesion mimic mutants that exhibit spontaneous cell death in the absence of pathogens [6]. One of the most important lesion mimic mutants is *lesions simulating disease resistance 1* (*lsd1*). The *lsd1* mutant exhibits runaway cell death when inoculated with avirulent bacterial pathogens, suggesting that AtLSD1 (AGI:At4g20380) is a negative regulator of PCD [7]. In addition, both superoxide and salicylic acid (SA) are necessary and sufficient to trigger cell death in the *lsd1* mutant [8], [9]. Genetic studies have indicated that *EDS1*, *PAD4*, and *NIM1/NPR1* are necessary for runaway cell death in the *lsd1* mutant [9], [10], whereas *AtrbohD* negatively regulates SA-triggered cell death in the *lsd1* mutant [11]. *AtLSD1* has been shown to negatively regulate reactive oxygen species (ROS) and stress-induced ethylene levels [8], [12], [13], [14], [15], [16]. AtLSD1 is a novel zinc finger protein containing three zinc finger domains and has been shown to interact with AtbZIP10 and AtMC1 [17], [18], [19].

The LITAF domain is named after the human LITAF [lipopolysaccharide (LPS)-induced tumor necrosis factor alpha (TNF-α) factor] protein. It contains an N-terminal CxxC knuckle, a hydrophobic region, and a C-terminal (H)xCxxC knuckle [20]. The LITAF domain exists in viruses, fungi, plants, and metazoa (http://www.ebi.ac.uk/interpro/IEntry?ac=IPR006629). Currently, among all LITAF domain proteins, only LITAF and SIMPLE (small integral membrane protein of the lysosome/late endosome) have been characterized. Human LITAF is a novel LPS-induced transcription factor involved in activating TNF-α gene expression [21]. It is notable that both LITAF and SIMPLE are encoded by the same gene *PIG7* (p53-induced gene 7) [22]. Direct and indirect evidence has suggested that *SIMPLE/LITAF/PIG7* is an important gene involved in human diseases such as Charcot-Marie-Tooth disease 1C (CMT1C) [23], [24], [25]. Moreover, *SIMPLE/LITAF/PIG7* is dramatically induced during p53-mediated apoptosis [26], suggesting that it may be involved in the regulation of PCD [20], [22]. However, the role of LITAF domain proteins in PCD has not been characterized.

In the present study, we report the role of plant LITAF domain protein GILP in the negative regulation of PCD. Our results show that AtGILP directly interacts with AtLSD1, the expression of *AtGILP* is induced by both *Pst avrRpt2* and FB1, and overexpression of *AtGILP* can suppress avirulent pathogen*-*triggered PCD. Thus, we present evidence that the plant LITAF domain protein GILP is a novel regulator of PCD.

## Results

### Identification of the plant LITAF domain protein GILP

**Figure 1:**
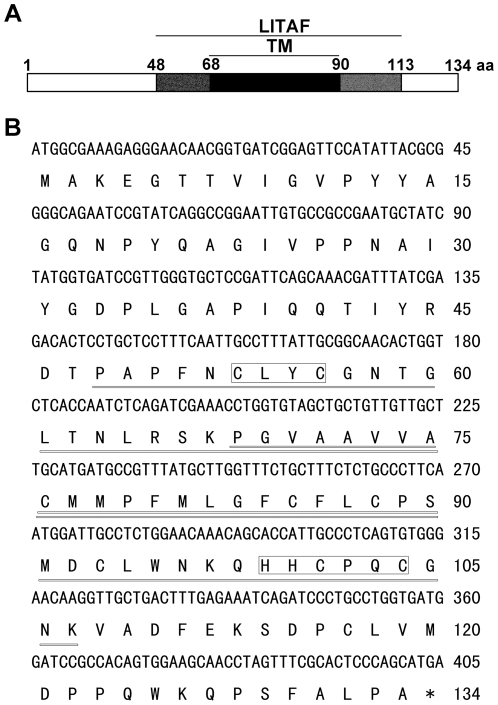
AtGILP gene structure and sequence. (A) Schematic diagram of the AtGILP amino acid sequence. Amino acids are numbered on the scale above the diagram. The central region of AtGILP contains a LITAF domain, which contains a putative transmembrane region (TM) in the central region. (B) AtGILP cDNA sequence and deduced amino acid sequence. The nucleotide and amino acid positions are shown on the right. The LITAF domain predicted by the SMART program (http://smart.embl-heidelberg.de/) is underlined. A putative transmembrane domain predicted by the TMHMM program (http://www.cbs.dtu.dk/services/TMHMM-2.0/) is double-underlined. The two knuckles are boxed.

Previously, we isolated a reduced glutathione (GSH)-induced pea cDNA encoding a LITAF domain protein and named it *Pisum sativa GSH-induced LITAF domain protein* (*PsGILP*; GenBank accession number: AAY40471.1). To analyze the function of GILP in *Arabidopsis*, we cloned the orthologous gene of *PsGILP* from *Arabidopsis*. *AtGILP* (AGI: At5g13190) encodes a protein of 134 amino acids with an estimated molecular mass of 14.56 kDa (Figure 1A and 1B). One LITAF domain was predicted by the SMART program and is located in the middle of the AtGILP protein between amino acids 48 and 113 (Figure 1A and 1B). TMHMM program analysis showed that a putative transmembrane region is located in the middle of the LITAF domain between amino acids 68 and 90 (Figure 1A and 1B). Moreover, the LITAF domain of AtGILP contains both an N-terminal CxxC knuckle and a C-terminal (H)xCxxC knuckle (Figure 1B).

BLAST program analysis showed that GILP is the only LITAF domain protein in plants. Sequence alignment analysis showed that the amino acid sequence of GILP is highly conserved in plants (Figure S1).

### AtGILP localizes in the plasma membrane

**Figure 2:**
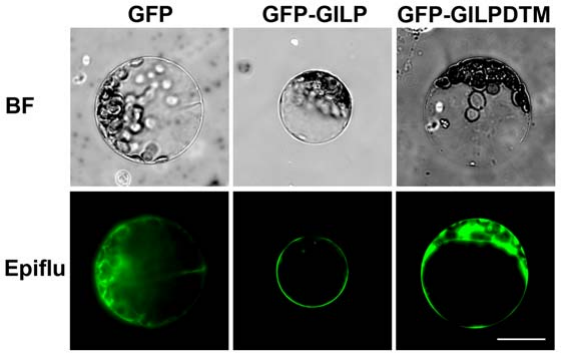
Plasma membrane localization of AtGILP. Plasmids expressing GFP (left panel), GFP-AtGILP (middle panel), or GFP-AtGILPΔTM (right panel) were transfected into Arabidopsis mesophyll protoplasts. Fluorescent images were taken at 12–16 h after transfection. BF and Epiflu indicate bright field and epifluorescence, respectively. The scale bar is 20 µm. Results shown are representative of three independent experiments.

As described above, the AtGILP protein contains a putative transmembrane region in the middle of the LITAF domain, suggesting its localization in the cell membrane (Figure 1). To determine the subcellular localization of AtGILP, we constructed a fusion of the green fluorescent protein (GFP) gene and *AtGILP* under the control of the CaMV 35S promoter and monitored the localization of GFP-AtGILP in *Arabidopsis* mesophyll protoplasts. As shown in Figure 2, GFP alone was distributed throughout the cytoplasm and the nucleus (left panel), whereas GFP-AtGILP was exclusively localized in the plasma membrane (middle panel). This result indicates that AtGILP localizes in the plasma membrane.

To further confirm the localization of AtGILP in the plasma membrane, we constructed a fusion of GFP and AtGILPΔTM, a truncated form of AtGILP with the transmembrane region (aa 68–90) deleted, driven by the CaMV 35S promoter. Similar to GFP, GFP-AtGILPΔTM was distributed in both the cytoplasm and the nucleus (Figure 2, right panel), indicating that the transmembrane region is necessary for AtGILP localization in the plasma membrane. Taken together, our findings suggest that AtGILP is localized in the plasma membrane and its plasma membrane localization depends on the transmembrane region of the LITAF domain.

### AtGILP interacts with AtLSD1

**Figure 3:**
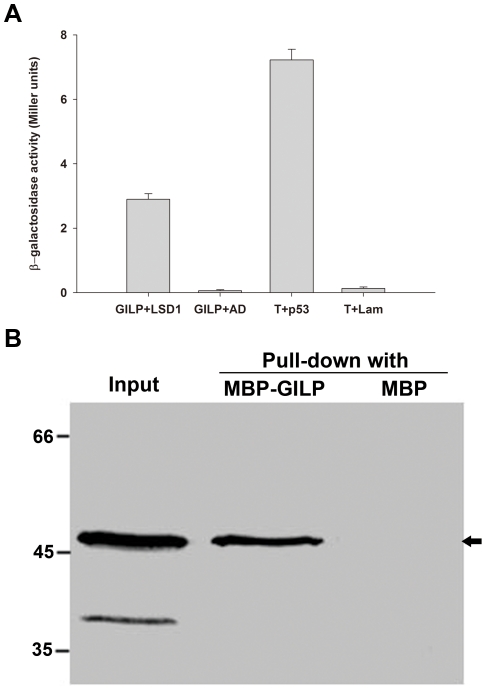
AtGILP interacts with AtLSD1. (A) AtGILP associates with AtLSD1 in yeast. pGBK-AtGILP was co-transformed with pGAD-AtLSD1 or pGADT7 (control) into yeast strain AH109 and β-galactosidase activity of the resulting colonies was measured. “T+p53” and “T+Lam” are positive and negative controls for the yeast two-hybrid assay, respectively. (B) AtGILP directly binds to AtLSD1 *in vitro*. Purified MBP-AtGILP or MBP (control) was incubated with GST-AtLSD1 supernatant and amylose agarose beads. Pulled-down proteins and “Input” sample (GST-AtLSD1 supernatant) were detected by Western blot using an anti-GST monoclonal antibody. Arrow indicates the GST-AtLSD1 protein band. All results shown are representative of two independent experiments.

Previously, we identified PsLSD1 (GenBank accession number: HQ006097) as a PsGILP-interacting protein in a yeast two-hybrid screen and confirmed the interaction between PsGILP and PsLSD1 in yeast (our unpublished data). This finding prompted us to test whether AtGILP interacts with AtLSD1. We initially performed yeast two-hybrid assays to test whether AtGILP interacts with AtLSD1. Quantitative β-galactosidase assays showed that AtGILP associated with AtLSD1 in yeast (Figure 3A). To confirm the interaction between AtGILP and AtLSD1, we carried out *in vitro* pull-down assay. Pull-down assays showed that AtGILP directly bound to AtLSD1 *in vitro* (Figure 3B). Taken together, our findings suggest that AtGILP interacts with AtLSD1.

### Both N-terminal and C-terminal domains of AtGILP interact with the N-terminal domain of AtLSD1

**Figure 4:**
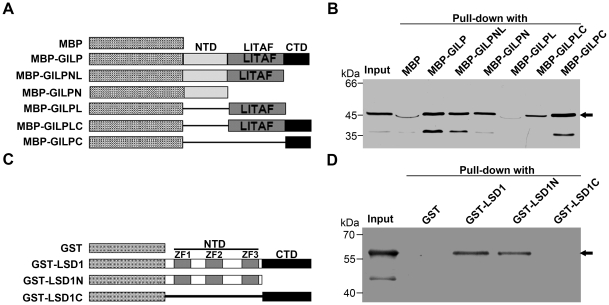
Domain mapping of the interaction between AtGILP and AtLSD1. (A) Schematic diagram of AtGILP mutants. LITAF, NTD, and CTD represent the LITAF domain, N-terminal domain, and C-terminal domain, respectively. (B) AtLSD1 interacts with both the N-terminal and the C-terminal domains of AtGILP. Purified MBP (control), MBP-AtGILP, MBP-AtGILPNL, MBP-AtGILPN, MBP-AtGILPL, MBP-AtGILPLC, or MBP-AtGILPC was incubated with GST-AtLSD1 supernatant and amylose agarose beads. Pulled-down proteins and “Input” sample (GST-AtLSD1 supernatant) were detected by Western blot using an anti-GST monoclonal antibody. Arrow indicates the GST-AtLSD1 protein band. (C) Schematic diagram of AtLSD1 mutants. zf1, zf2, and zf3 indicate the first, second, and third zinc finger domain, respectively. NTD and CTD represent N-terminal and C-terminal domain, respectively. (D) AtGILP interacts with the NTD of AtLSD1. GST (control), GST-AtLSD1, GST-AtLSD1N, or GST-AtLSD1C supernatant was incubated with purified MBP-AtGILP and glutathione agarose beads. Pulled-down proteins and “Input” sample (purified MBP-AtGILP) were detected by Western blot using an anti-MBP polyclonal antibody. Arrow indicates the MBP-AtGILP protein band.

Considering that the LITAF domain is located in the middle of AtGILP (Figure 1), we divided AtGILP into three domains: N-terminal domain (NTD, aa 1–47), LITAF domain (aa 48–113), and C-terminal domain (CTD, aa 114–134). To determine which domain of AtGILP is responsible for interacting with AtLSD1, we constructed a series of deletion mutants of AtGILP (Figure 4A) and tested their interaction with AtLSD1. Pull-down assay showed that both the N-terminal and the C-terminal domains of AtGILP were sufficient for the interaction with AtLSD1, whereas the LITAF domain was not involved in the interaction with AtLSD1 (Figure 4B).

Considering that the three zinc fingers are located in the N-terminal region of AtLSD1, we divided AtLSD1 into two domains and tested their interaction with AtGILP: N-terminal domain (NTD, aa 1 to 105) containing three zinc fingers and C-terminal domain (CTD, aa 106 to 176) (Figure 4C). Pull-down assay showed that the N-terminal domain, but not the C-terminal domain, of AtLSD1 could interact with AtGILP (Figure 4D). Taken together, our data suggest that AtGILP can interact with the N-terminal domain of AtLSD1 via its N-terminal or C-terminal domain.

### *AtGILP* expression is upregulated during both *Pst avrRpt2*- and FB1-induced PCD

**Figure 5:**
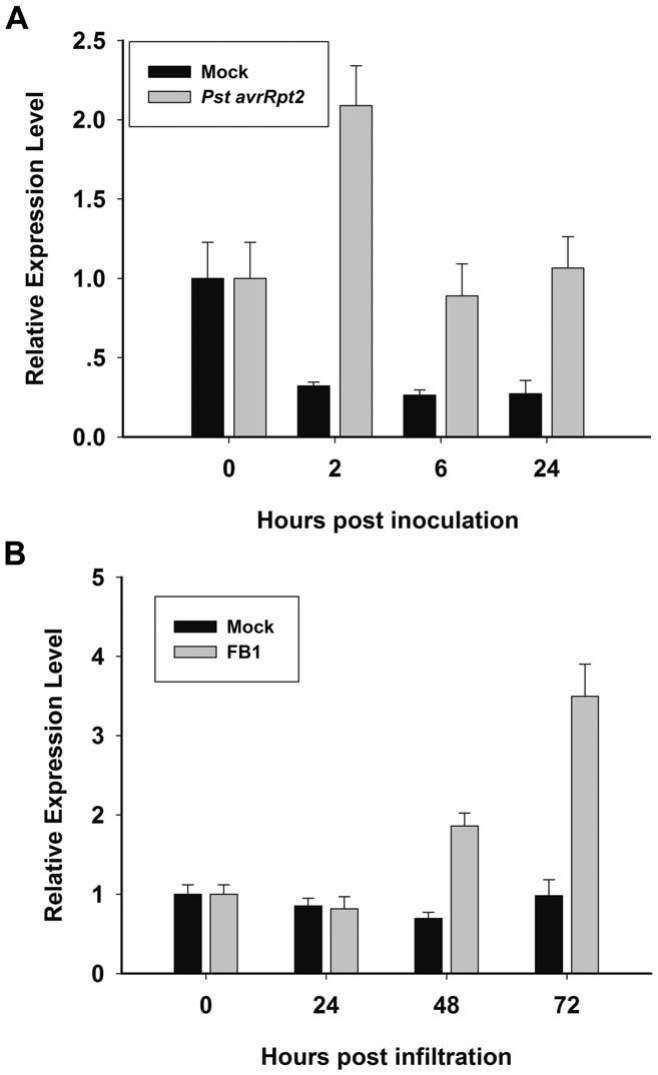
The expression of AtGILP is induced by both Pst avrRpt2 and FB1. (A)*AtGILP* is induced by *Pst avrRpt2*. Leaves of wild-type plants were inoculated with 10 mM MgCl2 (mock) or *Pst avrRpt2* (1×108 cfu/mL). (B) *AtGILP* is induced by FB1. Leaves of wild-type plants were infiltrated with 10 mM MgSO4 (mock) or 10 µM FB1. RNA was isolated at the indicated time points and analyzed by Real-time PCR. *AtUBQ10* was used as an internal control. Each data point consists of three replicates. Error bars indicate SD. The experiments were performed two times with similar results.

It has been shown that AtLSD1 is an important negative regulator of PCD [7]. The interaction between AtGILP and AtLSD1 suggests that AtGILP may be involved in the regulation of PCD. It is well known that avirulent pathogen *Pst avrRpt2* and fungal toxin fumonisin B1 (FB1) can trigger PCD in *Arabidopsi*s [27], [28]. To examine the role of AtGILP in the regulation of PCD, we treated five-week-old *Arabidopsis* with *Pst avrRpt2* and FB1 and analyzed whether the expression of *AtGILP* is affected during *Pst avrRpt2*- and FB1-induced PCD. Real-time PCR analysis showed that, compared with the mock treatment, *AtGILP* expression was significantly up-regulated at 2 h post-inoculation of *Pst avrRpt2* and at 72 h post-infiltration of FB1, respectively (Figure 5A and 5B). These results indicate that *AtGILP* is induced by both *Pst avrRpt2* and FB1.

### Overexpression of *AtGILP* suppresses RPS2-conditioned hypersensitive cell death

**Figure 6:**
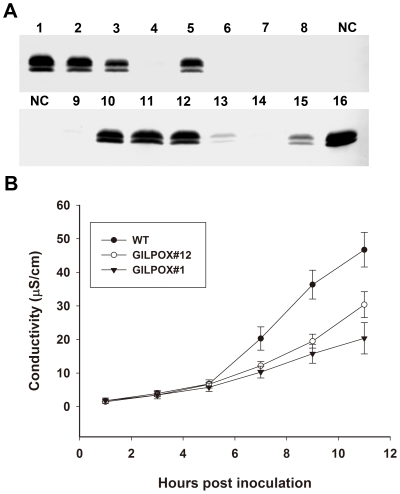
Overexpression of *AtGILP* suppresses RPS2-conditioned hypersensitive cell death. (A) Western blot analysis of transgenic lines using an anti-HA monoclonal antibody. Wild-type plants were used as the negative control. Various transgenic lines are numbered, and NC represents the negative control. (B) Quantification of cell death by electrolyte leakage assay. Plants of the indicated genotypes were inoculated with avirulent bacteria *Pst avrRpt2* at 5×107 cfu/mL and leaf discs were then excised to measure conductivity at the time points indicated. Each value represents the mean and SD of three replicates per experiment. The experiments were repeated three times with similar results.

The interaction of AtGILP with AtLSD1 and the induction of AtGILP expression by both *Pst avrRpt2* and FB1 suggested that AtGILP may function as a regulator of PCD. We next investigated the function of AtGILP in the regulation of PCD. Our initial effort to obtain GILP knockout or knockdown *Arabidopsis* plants proved to be unsuccessful. Therefore, we generated transgenic *Arabidopsis* plants overexpressing the AtGILP protein fused with an HA tag. We obtained several independent transgenic lines expressing high levels of the AtGILP protein by Western blot analysis (Figure 6A).

It is well-known that the plant R protein RPS2 can recognize the effector avrRpt2 harbored by the avirulent bacteria *Pst avrRpt2* to trigger the hypersensitive cell death in *Arabidopsis* [27]. Electrolyte leakage measurements have been extensively used to quantify the hypersensitive cell death [13], [29], [30], [31], [32], since cell death leads to electrolyte release, which is measured as changes in the conductivity of a bath solution [29]. To test whether AtGILP is involved in the regulation of PCD, we inoculated five-week-old plants with *Pst avrRpt2* and monitored electrolyte leakage to quantify the hypersensitive cell death. Compared with leaf discs from wild-type plants, those excised from transgenic lines #1 and #12 exhibited significantly lower ion leakage 5 hours after inoculation with *Pst avrRpt2* (Figure 6B). This result indicates that overexpression of *AtGILP* can inhibit pathogen-induced PCD.

## Discussion

One of the major challenges in elucidating the regulatory mechanism of hypersensitive cell death is the identification of novel regulators. In this study, we report that AtGILP, a LITAF domain-containing protein, is a novel negative regulator of hypersensitive cell death.

### The role of AtGILP and its human homolog in PCD

AtLSD1 is an important regulator of plant PCD [7], [8]. Our data demonstrated that AtGILP associates with AtLSD1 (Figure 3), implying that AtGILP may be involved in the regulation of PCD. Consistently, our data also showed that *AtGILP* is up-regulated during *Pst avrRpt2*- or FB1-induced PCD, providing further support for its role in the regulation of PCD (Figure 5). Moreover, over-expression of *AtGILP* inhibited *Pst avrRpt2*-induced hypersensitive cell death (Figure 6B), indicating that AtGILP is involved in the negative regulation of pathogen-induced PCD. Taken together, our data suggest that AtGILP is implicated in the negative regulation of hypersensitive cell death.

The human LITAF domain protein, SIMPLE, has been hypothesized to be involved in the regulation of PCD since it is dramatically upregulated during p53-mediated apoptosis [22], [26]. Consistent with this, *SIMPLE* has been identified as a novel candidate tumor suppressor gene since it is silenced by homozygous deletion or promoter hypermethylation in B-cell lymphoma [25]. In addition, GILP and SIMPLE are localized in the plasma membrane (Figure 2) [33]. Thus, we speculate that GILP and SIMPLE represent evolutionarily distant LITAF domain proteins with functions in regulating PCD. However, a direct role of SIMPLE in the regulation of PCD remains to be demonstrated.

### The plasma membrane localization of AtGILP

AtGILP contains a putative transmembrane region in the LITAF domain, suggesting its localization in the cell membrane (Figure 1). Subcellular localization analysis showed that AtGILP was localized in the plasma membrane (Figure 2, middle panel). This finding was confirmed by the observation that deletion of the putative transmembrane region caused AtGILP to lose its plasma membrane localization (Figure 2, right panel). Thus, our data demonstrate that AtGILP is localized in the plasma membrane. Similarly, another LITAF domain protein, SIMPLE, has been shown to colocalize with its interacting protein Nedd4 at the plasma membrane [33].

It is believed that plasma membrane proteins, such as BAK1, BIR1, and RING1, are involved in the regulation of PCD-related signaling pathways. BAK1 is a transmembrane receptor kinase involved in the negative regulation of PCD [34], [35]. The BAK1-interacting protein BIR1 is a receptor-like kinase involved in the negative regulation of PCD [36]. RING1 is a ubiquitin ligase involved in triggering FB1-induced PCD, and it may target degradation of negative regulators of PCD [37]. Considering that AtGILP interacts with AtLSD1 (Figure 3) and negatively regulates pathogen-induced PCD (Figure 6B), we postulate that AtGILP is involved in regulating PCD-associated signaling pathways.

### LITAF domain proteins may be membrane anchors

The importance of the LITAF domain has been suggested by findings that CMT1C-associated mutations of SIMPLE cluster in the LITAF domain [38], [39]. In the LITAF domain, the hydrophobic region between the N-terminal and C-terminal CxxC knuckles has been hypothesized to insert into, but not transverse, membranes and bring the two knuckles together to form a compact Zn^2+^-binding structure [20]. This hypothesis is supported by our findings that both the N-terminal and C-terminal domains of AtGILP interact with AtLSD1 (Figure 4B) and AtPILP1 (*Arabidopsis thaliana* Pirh2-like protein 1, AGI: At5g18650; Figure S2). Moreover, our data show that the transmembrane region of the LITAF domain is essential for the plasma membrane localization of AtGILP (Figure 2). Thus, the LITAF domain plays an essential role in membrane localization via insertion of its transmembrane region into membranes.

Because the LITAF domain of AtGILP is involved in plasma membrane localization, we assumed that the N-terminal or C-terminal domain of AtGILP may be involved in the protein–protein interaction. Interestingly, our results showed that both the N-terminal and the C-terminal domains of AtGILP, which do not have amino acid sequence similarities, were sufficient for the interaction with AtLSD1 (Figure 4B). Similarly, another AtGILP-interacting protein, AtPILP1, also interacted with both the N-terminal and the C-terminal domains of AtGILP (Figure S2). Thus, both the N-terminal and the C-terminal domains of AtGILP are involved in the protein–protein interaction. Consistent with our findings, the PPXY, PPSY, and P(S/T)AP motifs of SIMPLE, not the LITAF domain, are involved in the protein–protein interaction [33], [40], [41]. Taken together, these findings suggest that for LITAF domain proteins, the LITAF domain plays a role in membrane localization, whereas other domains are involved in the protein–protein interaction. These data suggest that LITAF domain proteins may act as membrane anchors by localizing in membranes via the LITAF domain, and they may bring other proteins to the membrane via protein–protein interaction domains.

In summary, our data suggest that AtGILP is localized in the plasma membrane, interacts with AtLSD1, and negatively regulates pathogen-induced PCD. Moreover, the LITAF domain plays an essential role in the plasma membrane localization of AtGILP, whereas both the N-terminal and the C-terminal domains are involved in the interaction of AtGILP with AtLSD1. Thus, our data suggest that AtGILP may act as a membrane anchor, bringing other regulators of PCD such as AtLSD1 to the plasma membrane.

## Materials and Methods

### Plant materials and growth conditions

All *Arabidopsis* plants were of the ecotype Columbia (Col-0) background. Seeds were soaked in 70% ethanol plus 0.01% Triton X-100 for 10 min and washed five times with sterile water. Surface-sterilized seeds were sowed on MS medium [4.3 g/L MS salts (Sigma, USA), 3% sucrose, 0.8% agar, pH 5.7]. After stratification at 4°C for 2–4 days, the plates were transferred to a tissue culture room at 22°C under a 9-h photoperiod. After seven days of growth, robust seedlings were potted in soil and grown in a growth chamber at 22°C and 60% relative humidity under a 9-h photoperiod.

### Subcellular localization

The coding region of AtGILP was amplified and introduced into pAVA121 [42] via *Bgl*II/*Xba*I to generate the GFP-AtGILP fusion construct. AtGILPΔTM was generated by recombinant PCR amplification and introduced into pAVA121 via *Bgl*II/*Xba*I to generate the GFP-AtGILPΔTM fusion construct. The primers used are listed in Table S1.

Protoplasts were prepared from well-expanded leaves of four- to five-week-old *Arabidopsis* plants and transfected with the GFP fusion constructs according to the protocol published by Yoo et al [43]. After incubation at room temperature for 12–16 h, the transfected protoplasts were visualized under a fluorescence microscope (Leica, Germany).

### Pathogen inoculation and FB1 treatment

Pathogen inoculation was performed essentially according to the protocol described by Katagiri et al [44]. Briefly, *Pst avrRpt2* was grown overnight at 28°C in the KB medium [45] with rifampicin (25 µg/mL) and kanamycin (50 µg/mL). Bacteria were collected, washed, and resuspended in 10 mM MgCl_2_. Five-week-old *Arabidopsis* leaves were infiltrated with bacteria or 10 mM MgCl_2_ (mock) using a 1 mL needleless syringe. For FB1 treatment, *Arabidopsis* leaves were infiltrated with 10 µM FB1 (in 10 mM MgSO_4_; Sigma, USA) or 10 mM MgSO_4_ (mock) using a 1 mL needleless syringe.

### Real-time PCR analysis

Total RNA was extracted from *Arabidopsis* leaves using TRIzol reagent (Invitrogen, USA) and treated with RNase-free DNase I (Takara, Japan). The first-strand cDNA was synthesized from DNase I-treated total RNA (1 µg) using the RevertAid^TM^ First Strand cDNA Synthesis Kit (Fermentas, Canada) and then diluted 15-fold to provide the template for real-time RT-PCR. Real-time RT-PCR analysis was carried out using SYBR Green real-time PCR Master Mix (TOYOBO, Japan) in the DNA Engine Opticon 2 Continuous Fluorescence Detector (MJ Research, USA). Data were analyzed using Opticon Monitor 2 software (MJ Research, USA). Relative expression levels for each target gene were calculated by the 2^-ΔΔCt^ method [46] using *AtUBQ10* as the internal control gene. The primers used are listed in Table S1.

### Yeast two-hybrid assay

The coding regions of AtGILP and AtLSD1 were amplified and cloned via *Eco*RI/*Sal*I into the bait vector pGBKT7 (Clontech, USA) and the prey vector pGADT7-Rec (Clontech, USA), respectively. Yeast transformation and β-galactosidase activity assay were performed as the manufacturer's protocols (Clontech, USA). The primers used are listed in Table S1.

### Recombinant protein expression and purification and *in vitro* pull-down assay

The coding regions of AtGILP and AtLSD1 were amplified and cloned via *Eco*RI/*Sal*I into the MBP-fusion expression vector pMAL-c2X (New England Biolabs, USA) and the GST-fusion expression vector pGEX-4T-1 (Amersham Biosciences, Sweden), respectively. Deletion mutants of AtGILP were generated by PCR amplification of the corresponding cDNA fragments and subsequently cloned via *Eco*RI/*Sal*I into pMAL-c2X. Deletion mutants of AtLSD1 were generated by PCR amplification of the corresponding cDNA fragments and subsequently cloned via *Eco*RI/*Sal*I into pGEX-4T-1. The primers used are listed in Table S1.

All constructs were transformed into *Escherichia coli* BL21 (DE3) cells. For batch purification of MBP fusion proteins, BL21 bacteria (100 mL culture volume) were collected and lysed by sonication in 10 mL of column buffer [20 mM Tris-HCl (pH 7.4), 200 mM NaCl, 1 mM EDTA, 1 mM phenylmethylsulphonyl fluoride (PMSF), 4 µg/mL aprotinin, 4 µg/mL leupeptin]. The lysates containing MBP fusion proteins were bound to 100 µL of amylose resins (New England Biolabs, USA), washed four times with 1 mL of column buffer, and eluted with 200 µL of column buffer containing 10 mM maltose. The concentration of purified MBP fusion proteins was determined by the Bradford method. For GST fusion proteins, BL21 bacteria (100 mL culture volume) were collected and lysed by sonication in 10 mL of pull-down binding buffer (20 mM Tris-HCl at pH 7.4, 150 mM NaCl, 1 mM EDTA, 1% Triton X-100, 1 mM PMSF, 4 µg/mL aprotinin, 4 µg/mL leupeptin). To prepare the GST fusion protein supernatant, the lysate was centrifuged at 12,000 rpm for 20 min at 4°C. The supernatant was immediately used for the *in vitro* pull-down assay.

For the *in vitro* pull-down assay, 500 µL of the GST fusion protein supernatant, 3 µg MBP fusion proteins, and 30 µL of amylose resins or glutathione-agarose resins (Sigma, USA) were incubated at 4°C for 4 h. The beads were then washed four times with 1 mL of wash buffer (20 mM Tris-HCl at pH 7.4, 500 mM NaCl, 1% Trition X-100) and eluted with 50 µL of 1× SDS sample buffer. For “Input” sample, 50 µL of the GST-AtLSD1 protein supernatant and diluted MBP-AtGILP protein (6 ng/µL) were mixed with 50 µL of 2× SDS sample buffer, respectively. Twenty microliters of pulled-down proteins and “Input” samples were separated on 10% SDS-PAGE and detected by Western blot using an anti-GST monoclonal antibody (for pulled-down proteins by amylose resins; Sigma, USA) or an anti-MBP polyclonal antibody (for pulled-down proteins by glutathione-agarose resins; New England Biolabs, USA).

### Construction of transgenic plant overexpressing AtGILP

A linker containing the HA tag was cloned into the plant expression vector pRTL2 [47] via *Nco*I/*Sac*I to generate pRTL-HA. AtGILP cDNA was amplified and cloned into pRTL-HA via *Sac*I/*Bgl*II to generate pRTL-HA-AtGILP. The expression cassette *35S:HA-AtGILP* was cleaved from pRTL-HA-AtGILP via *Pst*I and cloned into the *Pst* I site of pCAMBIA1381 to generate 1381-HA-AtGILP. The primers used are listed in Table S1.

The construct was introduced into *Agrobacterium* strain EHA105 by electroporation. *Agrobacterium* harboring the construct were used to transform wild-type Col-0 by the floral-dip method [48]. The resulting T_1_ transgenic seeds were screened in MS medium containing 25 µg/mL hygromycin and 100 µg/mL carbenicillin. Independent transformants were analyzed for the expression level of HA-AtGILP by Western blot using an anti-HA monoclonal antibody (Sigma, USA).

### Electrolyte leakage assay

Electrolyte leakage assay was performed essentially as previously described with minor modifications [31]. Five-week-old *Arabidopsis* leaves were inoculated with avirulent bacteria *Pst avrRpt2* in 10 mM MgCl_2_ using a 1 mL needleless syringe. Ten minutes after inoculation, four leaf discs (6 mm in diameter) were excised from four different plants of the same genotype, floated in 20 mL distilled water for 30 min, and then transferred to a 6-well tissue culture plate with each well containing 6 mL fresh distilled water. At the indicated time points, the conductivity of the solution was measured with a conductivity meter (Hanna, Italy).

## Supporting Information

Figure S1 **Multiple sequence alignment of GILPs.** The GILPs are shown in the following order (UniProtKB/TrEMBL accession number): *Ricinus communis* (*Rc*) GILP (B9RFX0), *Populus trichocarpa* (*Pt*) GILP (B9GMQ9), *Sorghum bicolor* (*Sb*) GILP (C5XT77), *Zea mays* (*Zm*) GILP (B6TNU5), *Oryza sativa* (*Os*) GILP (Q67UN6), *Arabidopsis thaliana* (*At*) GILP (Q94CD4), *Medicago truncatula* (*Mt*) GILP (B7FMI0), *Pisum sativum* (*Ps*) GILP (Q4U6G1), *Glycine max* (*Gm*) GILP (C6SYT8), and *Vitis vinifera* (*Vv*) GILP (A5ACU3). Amino acid sequences of GILPs were analyzed by the ClustalW2 program (http://www.ebi.ac.uk/Tools/clustalw2/index.html). The LITAF domain is underlined.
(TIF)

Figure S2 **Both the N-terminal and the C-terminal domains of AtGILP are involved in the interactions with AtPILP1.** (A) Schematic diagram of AtGILP mutants. LITAF, NTD, and CTD represent the LITAF domain, N-terminal domain, and C-terminal domain, respectively. BD represents the DNA binding domain of GAL4. (B)Both the N-terminal and the C-terminal domains of AtGILP interact with AtPILP1. pGBKT7 (control), pGBK-AtGILP, pGBK-AtGILPN, pGBK-AtGILPL, and pGBK-AtGILPC were co-transformed with pGAD-AtPILP1 into yeast strain AH109 respectively, and β-galactosidase activity of the resulting clones was measured. To generate the construct pGBK-AtGILPN, pGBK-AtGILPL, and pGBK-AtGILPC, the coding regions of the N-terminal, LITAF, and C-terminal domains of AtGILP were cleaved from the constructs MBP-AtGILPN, MBP-AtGILPL, and MBP-AtGILPC via *Eco*RI/S*al*I and cloned into the bait vector pGBKT7 (Clontech, USA), respectively. To generate the construct pGAD-AtPIPL1, the coding region of AtPIPL1 was amplified and cloned via *Bam*HI/*Sal*I into the prey vector pGADT7-Rec (Clontech, USA). The primers used are listed in Table S1.
(TIF)

Table S1 **Primers used in this study.**
(DOC)
