# Correction: The LSD1-Interacting Protein GILP Is a LITAF Domain Protein That Negatively Regulates Hypersensitive Cell Death in *Arabidopsis*

**DOI:** 10.1371/annotation/64462bfc-a7c6-48e2-9710-8ecea202e4a8

**Published:** 2011-06-30

**Authors:** Shanping He, Guihong Tan, Qian Liu, Kuowei Huang, Jiao Ren, Xu Zhang, Xiangchun Yu, Ping Huang, Chengcai An

Several grant numbers are incorrect in the funding information section. The correct funding information is: "This work was supported by grants from the National Key Basic Science "973" Program (2011CB016141), the National Natural Science Foundation (90817001 and 30671402), the National Special Projects for R&D of Transgenic Plants (2008ZX08009-004 and 2008ZX08010-001), and the State "863" High Technology R&D Program (2006AA06Z341) of the Chinese Government. The funders had no role in study design, data collection and analysis, decision to publish, or preparation of the manuscript."

