# Supplementary figures and images for: The LSD1-Interacting Protein GILP Is a LITAF Domain Protein That Negatively Regulates Hypersensitive Cell Death in Arabidopsis
Source: PLoS One. 2011 Apr 19;6(4):e18750. doi: 10.1371/journal.pone.0018750 (PMC3079718; doi:10.1371/journal.pone.0018750)

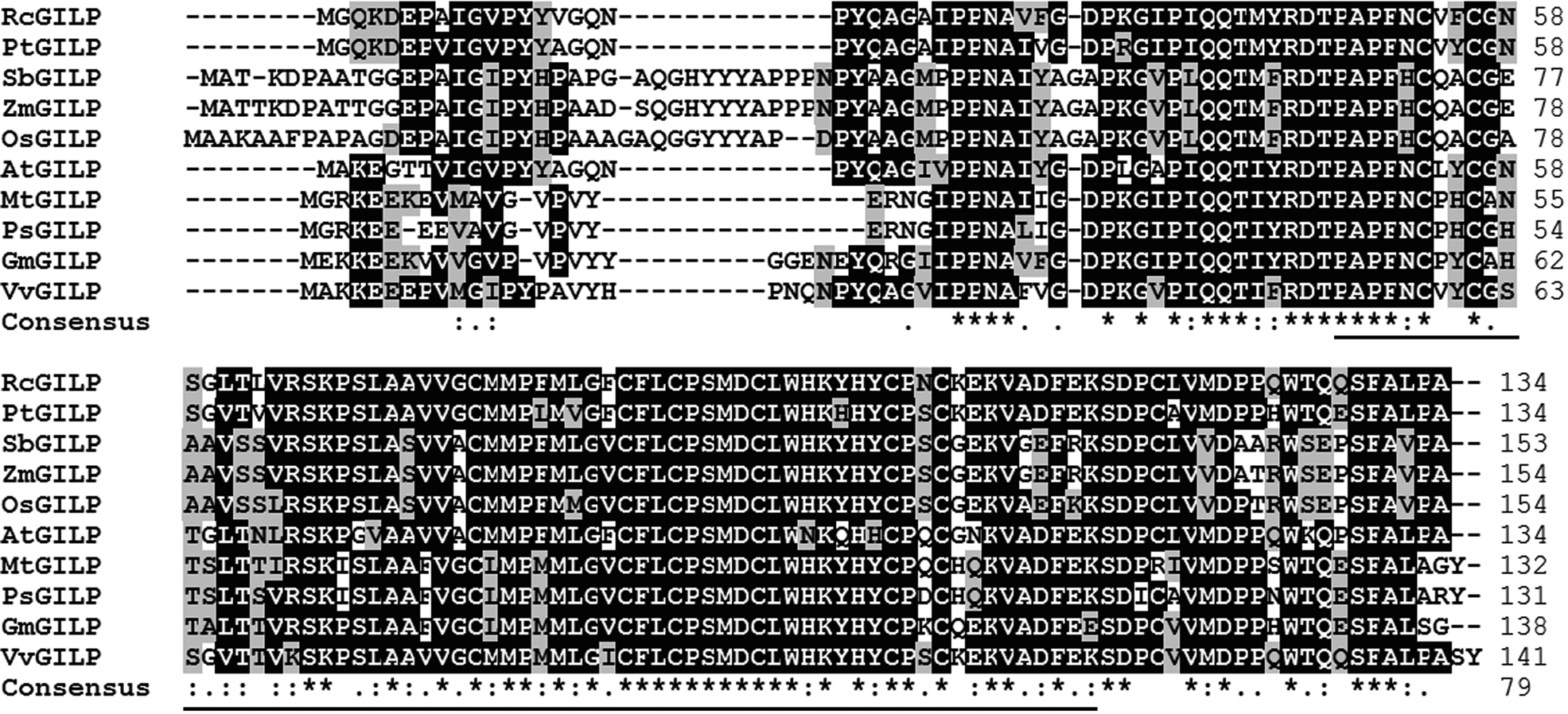

Supplement: Figure S1 — Multiple sequence alignment of GILPs. The GILPs are shown in the following order (UniProtKB/TrEMBL accession number): Ricinus communis (Rc) GILP (B9RFX0), Populus trichocarpa (Pt) GILP (B9GMQ9), Sorghum bicolor (Sb) GILP (C5XT77), Zea mays (Zm) GILP (B6TNU5), Oryza sativa (Os) GILP (Q67UN6), Arabidopsis thaliana (At) GILP (Q94CD4), Medicago truncatula (Mt) GILP (B7FMI0), Pisum sativum (Ps) GILP (Q4U6G1), Glycine max (Gm) GILP (C6SYT8), and Vitis vinifera (Vv) GILP (A5ACU3). Amino acid sequences of GILPs were analyzed by the ClustalW2 program (http://www.ebi.ac.uk/Tools/clustalw2/index.html). The LITAF domain is underlined. (TIF) [file pone.0018750.s001.tif]

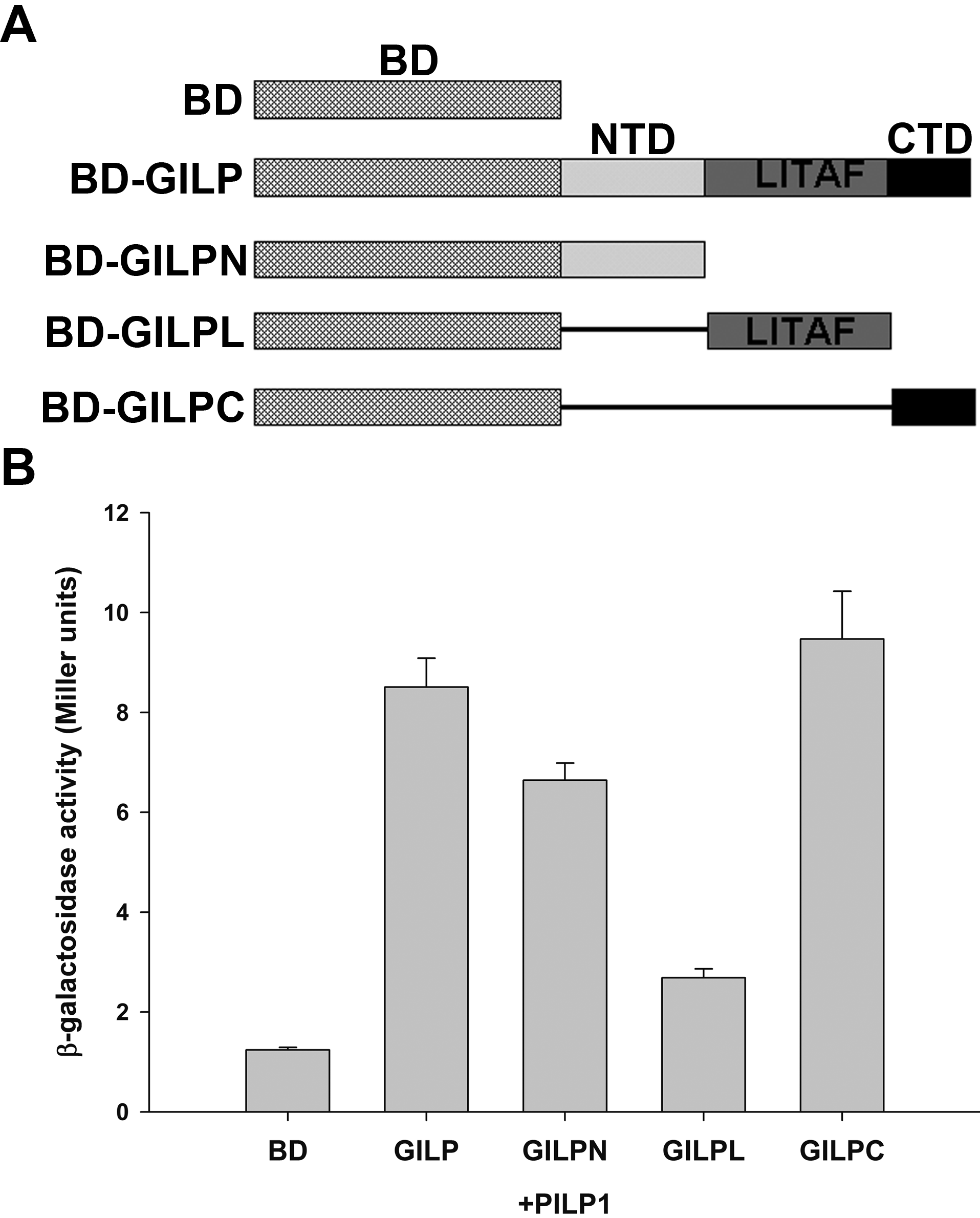

Supplement: Figure S2 — Both the N-terminal and the C-terminal domains of AtGILP are involved in the interactions with AtPILP1. (A) Schematic diagram of AtGILP mutants. LITAF, NTD, and CTD represent the LITAF domain, N-terminal domain, and C-terminal domain, respectively. BD represents the DNA binding domain of GAL4. (B)Both the N-terminal and the C-terminal domains of AtGILP interact with AtPILP1. pGBKT7 (control), pGBK-AtGILP, pGBK-AtGILPN, pGBK-AtGILPL, and pGBK-AtGILPC were co-transformed with pGAD-AtPILP1 into yeast strain AH109 respectively, and β-galactosidase activity of the resulting clones was measured. To generate the construct pGBK-AtGILPN, pGBK-AtGILPL, and pGBK-AtGILPC, the coding regions of the N-terminal, LITAF, and C-terminal domains of AtGILP were cleaved from the constructs MBP-AtGILPN, MBP-AtGILPL, and MBP-AtGILPC via EcoRI/SalI and cloned into the bait vector pGBKT7 (Clontech, USA), respectively. To generate the construct pGAD-AtPIPL1, the coding region of AtPIPL1 was amplified and cloned via BamHI/SalI into the prey vector pGADT7-Rec (Clontech, USA). The primers used are listed in Table S1. (TIF) [file pone.0018750.s002.tif]
