# Supplementary material for: The LSD1-Interacting Protein GILP Is a LITAF Domain Protein That Negatively Regulates Hypersensitive Cell Death in Arabidopsis
Source: PLoS One. 2011 Apr 19;6(4):e18750. doi: 10.1371/journal.pone.0018750 (PMC3079718; doi:10.1371/journal.pone.0018750)
Supplement: Table S1 — Primers used in this study. (DOC) [file pone.0018750.s003.doc]

**Table S1. Primers used in this study.**

| **Primer name** | **Sequence** | **Function** |
| --- | --- | --- |
| GILP-S | 5’-GCGCGAATTCAAGGAGATGGCGAAAGAGGG-3’ | pGBK-GILP  c2X-GILP |
| GILP-A | 5’-GCGCGTCGACTTATCATGCTGGGAGTGCGA-3’ |
| GILP-Bgl | 5’-GCGCAGATCTAAGGAGATGGCGAAAGAGGG-3’ | GFP-GILP |
| GILP-Xba | 5’-GCGCTCTAGATTATCATGCTGGGAGTGCGA-3’ |
| GILP-DS | 5’-CGAAAATGGATTGCCTCTGGAACAA-3’ | GFP-GILP△TM |
| GILP-DA | 5’-TCCATTTTCGATCTGAGATTGGTGA-3’ |
| LSD1-S | 5’-GCGCGAATTCTTTGTGTGTGTTTGGATGAA-3’ | pGEX-LSD1 |
| LSD1-A | 5’-GCGCGTCGACAAGATCTCACTCATTCTTGA-3’ |
| GILP-S1 | 5’- GCGCGAATTCGACACTCCTGCTCCTTTCAA -3’ | c2X-GILPNL  c2X-GILPN  c2X-GILPL  c2X-GILPLC  c2X-GILPC |
| GILP-A1 | 5’-GCGCGTCGACTTATCTGATTTCTCAAAGTCAGC-3’ |
| GILP-S2 | 5’- GCGCGAATTCGAGAAATCAGATCCCTGCCT-3’ |
| GILP-A2 | 5’-GCGCGTCGACTTAAGTGTCTCGATAAATCGTTT-3’ |
| LSD1-S1 | 5’- GCGCGAATTCGTTAATATGAGCAATGGAAG-3’ | pGEX-LSD1N  pGEX-LSD1C |
| LSD1-A1 | 5’-GCGCGTCGACAGTTACGAATTGACAAACAG-3’ |
| GILP-QS | 5’-GAGACACTCCTGCTCCTTTCA-3’ | Realtime PCR |
| GILP-QA | 5’-AACCAAGCATAAACGGCATC -3’ |
| UBQ10-S | 5’ -TCTAAATCTCGTCTCTGTTATGCTT -3’ | Realtime PCR |
| UBQ10-A | 5’- TAACAGGAACGGAAACATAGTAGAA -3 |
| HA-S | 5’-CATGGACTACCCATACGATGTTCCTGACTATGCGGACT-3’ | HA tag |
| HA-A | 5’-CCGCATAGTCAGGAACATCGTATGGGTAGTC-3’ |
| GILP-Sac | 5’-GCGCGAGCTCAAGGAGATGGCGAAAGAGGG-3’ | RTL-HA-GILP |
| GILP-Bgl | 5’- GCGCAGATCTTTATCATGCTGGGAGTGCGA-3’ |
| PILP1-S | 5’-GCGCGGATCCCTCATGGAAGCTTCACCCAA-3’ | pGAD-PILP1 |
| PILP1-A | 5’-GCGCGTCGACTGGTTTTGTCATTGAGGAAG-3’ |

Note: The restriction enzyme recognition sites are underlined.
